# WALANT versus intravenous regional anesthesia for carpal tunnel syndrome: a randomized clinical trial

**DOI:** 10.1590/1516-3180.2020.0583.R2.0904221

**Published:** 2021-10-11

**Authors:** Aldo Okamura, Vinicius Ynoe de Moraes, Marcela Fernandes, Jorge Raduan-Neto, João Carlos Belloti

**Affiliations:** I MD. Doctoral Student and Hand Surgeon, Discipline of Hand and Upper Limb Surgery, Universidade Federal de São Paulo (UNIFESP), São Paulo (SP), Brazil; and Hand Surgeon, Hand Surgery Service, Hospital Alvorada Moema, United Health, São Paulo (SP), Brazil.; II MD, PhD. Hand Surgeon, Discipline of Hand and Upper Limb Surgery, Universidade Federal de São Paulo (UNIFESP), São Paulo (SP), Brazil; and Hand Surgeon, Hand Surgery Service, Hospital Alvorada Moema, United Health, São Paulo (SP), Brazil.; III MD, PhD. Hand Surgeon, Discipline of Hand and Upper Limb Surgery, Universidade Federal de São Paulo (UNIFESP), São Paulo (SP), Brazil; and Hand Surgeon, Hand Surgery Service, Hospital Alvorada Moema, United Health, São Paulo (SP), Brazil.; IV MD, PhD. Hand Surgeon, Discipline of Hand and Upper Limb Surgery, Universidade Federal de São Paulo (UNIFESP), São Paulo (SP), Brazil; and Hand Surgeon, Hand Surgery Service, Hospital Alvorada Moema, United Health, São Paulo (SP), Brazil.; V MD, MSc, PhD. Full Professor, Discipline of Hand and Upper Limb Surgery, Universidade Federal de São Paulo (UNIFESP), São Paulo (SP), Brazil; and Full Professor, Hand Surgery Service, Hospital Alvorada Moema, United Health, São Paulo (SP), Brazil.

**Keywords:** Carpal tunnel syndrome, Clinical trial [publication type], Anesthesia, local, Effectiveness, General surgery, Median nerve, Bier’s block, Comparative study, Compressive syndrome

## Abstract

**BACKGROUND:**

There are several anesthetic techniques for surgical treatment of carpal tunnel syndrome (CTS). Results from this surgery using the “wide awake local anesthesia no tourniquet” (WALANT) technique have been described. However, there is no conclusive evidence regarding the effectiveness of the WALANT technique, compared with the usual techniques.

**OBJECTIVE:**

To evaluate the effectiveness of the WALANT technique, compared with intravenous regional anesthesia (IVRA; Bier’s block), for surgical treatment of CTS.

**DESIGN AND SETTING:**

Randomized clinical trial, conducted at Hospital Alvorada Moema and the Discipline of Hand Surgery, Universidade Federal de São Paulo (UNIFESP), São Paulo (SP), Brazil.

**METHODS:**

Seventy-eight patients were included. The primary outcome was measurement of perioperative pain through a visual analogue scale (VAS). The secondary outcomes were the Boston Questionnaire score, Hospital Anxiety and Depression Scale (HADS) score, need for use of analgesics, operating room times, remission of paresthesia, failures and complications.

**RESULTS:**

The WALANT technique (n = 40) proved to be superior to IVRA (n = 38), especially for controlling intraoperative pain (0.11 versus 3.7 cm; P < 0.001) and postoperative pain (0.6 versus 3.9 cm; P < 0.001). Patients spent more time in the operating room in the IVRA group (59.5 versus 46 minutes; P < 0.01) and needed to use more analgesics (10.8 versus 5.7 dipyrone tablets; P = 0.02). Five IVRA procedures failed (5 versus 0; P = 0.06).

**CONCLUSIONS:**

The WALANT technique is more effective than IVRA for CTS surgery.

## INTRODUCTION

In most countries, surgical treatment of carpal tunnel syndrome (CTS) is usually performed in hospitals, using the open (classical) surgical technique that is preferred by specialists.^1^^,^^2^^,^^3^ Anesthetic technique preferences vary among surgeons. Intravenous regional anesthesia (IVRA), as described by Bier, is in widespread use: it is the second most popular technique among American specialists and the most popular in Brazil.^4^^,^^5^ However, over the last decade, performance of this surgery using the “wide awake local anesthesia no tourniquet” (WALANT) technique has been described. This has proven to be a safe procedure with lower costs.^6^^,^^7^^,^^8^

Recent studies have compared surgical outcomes from CTS treatments, including the costs of WALANT versus general anesthesia; local anesthesia with adrenaline in association with sedation; and tourniquet application with monitored anesthetic care (MAC) and intravenous sedation. The conclusion from these studies was that local anesthesia was more effective and presented lower cost.^9^^,^^10^^,^^11^ However, in evaluating the quality of evidence, we noticed that there was still a need for level I studies on this topic.

## OBJECTIVE

The aim of this study was to randomly evaluate the effectiveness of two anesthesia methods for CTS: the WALANT technique and the IVRA technique.

## METHODS

### Ethics

This study was approved under institutional review board (IRB) number 61597316.4.0000.5505 on November 28, 2016. The trial protocol was registered *a priori* under the number NCT02986347 (http://clinicaltrials.gov).

### Study design and setting

This was a randomized clinical trial with parallel groups (allocation ratio 1:1). It was conducted in accordance with the CONSORT statement for trial reporting. The study was conducted at two patient recruitment centers: Hospital Alvorada Moema and the Discipline of Hand Surgery, Universidade Federal de São Paulo (UNIFESP), São Paulo, Brazil.

### Inclusion criteria

Adult patients presenting with idiopathic CTS without prior hand surgery were included in this study. The diagnosis made through clinical evaluation and confirmed by means of electromyography.

A - The clinical criteria for diagnosing CTS were the presence of at least four of the following criteria, in accordance with CTS-6.^12^

1. Paresthesia in the territory of the median nerve.
2. Night paresthesia of the hand.
3. Decreased hand strength with thenar muscle hypotrophy.
4. Positive Tinel’s sign at the wrist.
5. Positive Phalen test.
6. Loss of two-point discrimination, greater than 6 mm.

B - The indications for surgical treatment were either of the following:

1. Failure of conservative treatment for at least three months, use of night splint and one local corticosteroid injection.
2. Motor impairment detected through clinical examination and proven by means of an electromyographic test. The criteria that we used were the presence of sensory and motor involvement, stratified as moderate or severe CTS, as described by Padua.^13^

C - Patients were included if their pre-anesthesia evaluation categorized them as ASA I or II, in accordance with the American Society of Anesthesiologists (ASA) classification.

### Exclusion criteria

The following individuals were excluded from this study:

1. Pregnant and postpartum women.
2. Patients who refused the terms of the research consent statement.
3. Patients who declared that they had previously undergone hand or wrist surgery.

### Intervention

**Figure 1.**
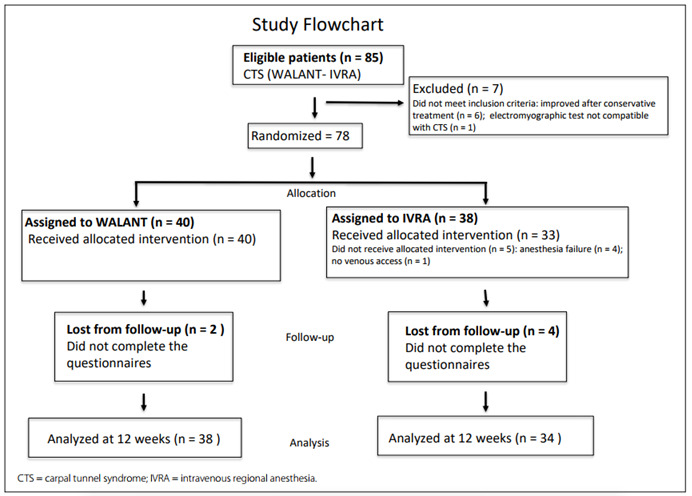
Study flowchart.

Out of the 85 patients eligible for this study, 78 were included and 72 (WALANT: 38; IVRA: 34) completed the 12-week follow-up. Four IVRA failures were found. The losses were balanced between the groups (WALANT, two losses; IVRA, four losses) **(**Figure 1**)**.

### Preoperative care

The participants in both groups were admitted to the hospital approximately two hours before the surgical procedure and intravenously received 100 mg ketoprofen and 500 mg dipyrone diluted in 250 ml of saline solution in the contralateral limb, for preemptive analgesia. In patients with known sensitivity to these drugs, tenoxicam (20 mg) and tramadol (50 mg) were used as substitutes.

In the surgical suite, all patients were adequately positioned in the supine position, with a cardiac monitor and noninvasive blood pressure and pulse oximetry. No prophylactic antibiotic was used.

### Intervention

#### WALANT technique

Anesthesia was administered by one of three hand surgery specialists who were already familiar with the WALANT technique.^5^ At the time of admission to the surgical suite, each patient received a 10 ml to 15 ml infusion of an anesthetic solution. This solution was composed of 1% lidocaine, with epinephrine at proportions of 1:100,000.

Initially, 5 ml of the solution was slowly applied in the wrist flexion crease region between the median and ulnar nerves, just below the skin and subfascial plane. The needle was moved slowly while observing the swelling of the tissues, and was redirected to the radial side of the proximal palmar region for injection of another 2-3 ml of the solution into the subcutaneous layer. The remaining 3-7 ml was injected into the subdermal plane, anteriorly to the transverse carpal ligament. The approximate time taken for the injection was about five minutes, with care to keep the needle at a margin of 5 mm from the already anesthetized region. During infiltration of the solution, swelling of the tissues and pallor of the skin were observed. No exsanguination or tourniquet of the limb was used.

### IVRA technique - intravenous regional anesthesia (Bier’s block)

The IVRA technique was performed by the anesthesia team in accordance with to the following procedure: 1) Venous puncture and catheterization with a catheter as distal as possible in the limb to be operated; 2) Exsanguination with an elastic Esmarch bandage from distal to proximal; 3) A second Esmarch bandage was placed in the proximal portion of the arm; 4) Slow injection of 40 ml of lidocaine without epinephrine at 0.5% (maximum 3-4 mg/kg); 5) After injection, the arm was lowered to the level of the table and the intravenous cannula was removed; 6) The tourniquet was removed after the end of the surgery, and 30 minutes after injection of the anesthetic; 7) Removal of the tourniquet was performed slowly, while maintaining serial ischemic subocclusions, totaling three minutes for the procedure.^14^

### Carpal tunnel syndrome surgery

Both groups underwent treatment using the same surgical technique. The surgery was performed by a hand surgery team with more than 10 years of surgical experience. A longitudinal palmar incision was made, distal to the wrist flexion plantar fold, centered on the cubital edge of the ring finger in flexion, of length around 5 cm, followed by dissection plane by plane until all the transverse carpal ligament and proximal forearm fascia had been sectioned, followed by inspection of the carpal tunnel. We did not perform routine neurolysis.

### Postoperative analgesia

During the in-hospital period, the participants in both groups received analgesia in accordance with the following protocol:

- Mild pain (up to 2.0 visual analogue scale [VAS] points): no medication.
- Moderate pain (between 2.1 and 5.0 VAS points): 500 mg of dipyrone intravenously, with a maximum dose of 4000 mg/day.
- Severe pain (5.1 to 9.0 VAS points): 50 mg of tramadol intravenously, with a maximum dose of 400 mg/day.
- Extreme pain (9.1-10 VAS points): 2 mg of morphine, with a maximum dose of 60 mg/day.

The patients were discharged 12 hours after the end of surgery.

### Outcomes

The outcome assessments were performed by a blinded researcher who was not directly linked to the study. These evaluations took place within the following timeframes:

- Office visit prior to surgery, on the date of scheduling the surgery.
- Postoperative period: 15 minutes after tourniquet release, 2 hours, 4 hours, 6 hours, 8 hours, 12 hours, 7 days, 14 days, 1 month and 3 months.

### Primary outcome

**Pain assessment** was done through a visual analogue scale (VAS).^15^ Pain was measured preoperatively and in the immediate postoperative period: 2 hours, 4 hours, 6 hours, 8 hours and 12 hours after completion of the surgery. The patients were instructed to measure their own pain level using the VAS. The score is determined by measuring the distance (mm) on the 100 mm line between the “painless” anchor and the patient’s indication, thus providing scores from 0 to 100. We considered that differences between measurements of more than two points were clinically relevant.^15^

### Secondary outcomes

The following secondary outcomes were measured:

**Use of analgesics** - From hospital discharge until the patient’s first return visit (seventh postoperative day), the number and type of analgesic drugs used by patients were recorded in relation to previous orientation.

**Anxiety and depression** - The Hospital Anxiety and Depression Scale (HADS) score^16^^,^^17^ was assessed at the time of patient admission and at the first outpatient return (seventh postoperative day). HADS consists of 14 self-reported questions: seven of them referring to anxiety (HADS-A) and seven to depression (HADS-D). The scores range from 0 to 21 points for each subscale and a score of 9 or higher defines a likely diagnosis of anxiety and depression. With this cutoff point, the instrument presents sensitivity for anxiety of 93.7% and for depression of 84.6%; and specificity for anxiety of 72.6% and for depression, of 90.3%.^17^

**Self-reported function** - The Boston Carpal Tunnel Questionnaire (BCTQ)^18^^,^^19^ was used for evaluations both preoperatively and postoperatively (three months). This is a disease-specific questionnaire for CTS that is self-administered. It evaluates the disease on two subscales: 1) severity of symptoms (SSS); and 2) functional status of patients (FSS) with CTS.

**Paresthesia remission** - After the surgical procedure and at the end of the first week and the third month of follow-up, an evaluation was made to verify remission of the paresthesia that patients had complained of preoperatively.

**Complications and failures** - All clinical events that occurred due to anesthesia and which required additional interventions not foreseen in this protocol were considered to be complications. We considered that failure of anesthesia had occurred when there was a need to change the anesthetic technique to which the patient had been allocated or when there was a need for surgical reintervention within the first three months after surgery. Patients who at some time presented complications or failures were given the usual necessary treatment and their results were computed within the group to which that had initially been allocated.

**Sample calculation** - We aimed to detect a minimum difference of two points (standard deviation, SD, of three points) on the VAS scale. This specification was derived from a systematic review that indicated that the minimum clinically important difference for VAS scales ranged from 0.8 to 4.0.^20^ We considered an 80% power and alpha of 5%. Considering also an attrition loss of 10%, we derived a sample size of 78 patients for inclusion in the study.

**Randomization and allocation** - The randomization sequence was generated by means of software (http://www.randomizer.org). The allocation was performed using 78 opaque sealed envelopes marked only with numbering. These were opened by a person not directly involved in the study. Each envelope was only opened after a patient entered the surgical suite.

**Statistical methods** - We presented the data as means and standard deviations and proportions. As a method for confirming the effectiveness of randomization, the baseline data were compared when stratified according to the allocation group. The assumption of normality of the distribution was made by applying the Shapiro-Wilk test and by visual judgment. The chi-square test was used to analyze the results from both groups in relation to the categorical variables. Student’s t test (parametric) or the Mann-Whitney U test was used to compare the groups in relation to the continuous variables. The significance level was set at 5%. The analysis was done in accordance with intention-to-treat principles.

## RESULTS

The sample consisted mostly of women (97.2%), with a mean age of 51 years. The patients had had their disease for a mean time of 4.5 years and most cases were considered to be moderate, as staged using electromyography. Additional baseline data demonstrated that the randomization methods presented adequate performance (Table 1), which thus resulted in balanced groups.

**Table 1.** Baseline data

Variable	WALANT (n = 38)	IVRA (n = 34)	P-value
Age (mean, SD)	51.6 (10.7)	51 (12)	0.84
Gender, female, n (%)	38 (100)	32 (94.1)	0.23
Affected side, n (%)	23 (60.5)	21 (61.7)	0.91
Dominant side, n (%)	36 (94.7)	32 (94.1)	0.90
Time with symptoms, years, (mean, SD)	5.3 (4.8)	4.4 (3.4)	0.77
Number of clinical criteria (mean, SD)	4.5 (0.7)	4.1 (0,5)	0.06
Electromyography, severe, n (%)	20 (52.6)	20 (58.8)	0.82
BCTQ pre (symptoms) (mean, SD)	37.3 (6.3)	37.7 (8.7)	0.81
BCTQ pre (function) (mean, SD)	25.1 (6.3)	24 (6.9)	0.49
Preoperative (mean, SD)	5.8 (2.8)	6 (2.1)	0.8
HADS (A) pre (mean, SD)	4.6 (3.5)	3.5 (3)	0.2

IVRA = intravenous regional anesthesia; SD = standard deviation; pre = preoperative; BCTQ = Boston Carpal Tunnel Questionnaire; HADS (A) = Hospital Anxiety and Depression Scale - anxiety subscale.

### Pain (VAS)

There were statistical differences between the groups at the following times: transoperative period, immediate postoperative period, 2 hours, 4 hours, 6 hours and 8 hours. Statistical differences with clinical relevance (> 2 VAS points) occurred in the immediate postoperative period and 2 hours after surgery (Table 2).

**Table 2.** Pain scores

Pain (VAS)	WALANT (n = 38)	IVRA (n = 34)	P-value
Preoperative (mean, SD)	5.8 (2.8)	6 (2.1)	0.8
Postoperative, immediate (mean, SD)	0.11 (0.7)	3.7 (3.9)	< 0.001^**^
Postoperative, 2 hours (mean, SD)	0.6 (1.8)	3.9 (2.4)	< 0.001^**^
Postoperative, 4 hours (mean, SD)	1 (2.2)	2.9 (2)	< 0.001^*^
Postoperative, 6 hours (mean, SD)	1.7 (2.1)	2.7 (2.1)	0.02^*^
Postoperative, 8 hours (mean, SD)	1.35 (1.9)	2.2 (1.8)	0.01^*^
Postoperative, 12 hours (mean, SD)	2 (2.2)	2.5 (2.2)	0.24

^*^Statistically significant (P < 0.05); ^**^with clinical relevance.

VAS = visual analogue scale; IVRA = intravenous regional anesthesia; SD = standard deviation.

### Operating room times

The duration of surgery for the WALANT group was 12.8 ± 3.8 minutes, while for the IVRA group it was 11 ± 3.2 minutes (P = 0.02). The WALANT group remained in the operating room for 46 ± 5.7 minutes, while the IVRA group was there for 59.5 ± 6.8 minutes (P < 0.01) (Table 3).

**Table 3.** Secondary outcomes

Variable	WALANT (n = 40)	IVRA (n = 38)	P-value
Operating room time, minutes (mean, SD)	46 (5.7)	59.5 (6.8)	< 0.01^*^
Surgery time, minutes (mean, SD)	12.8 (3.8)	11 (3.2)	0.02^*^
Dipyrone (n) (mean, SD)	5.7 (9.8)	10.8 (9.8)	0.02^*^
Tramadol (n) (mean, SD)	1.6 (3.3)	4 (8)	0.066
BCTQ pre (function) (mean, SD)	25.1 (6.3)	24 (6.9)	0.49
BCTQ three months (symptoms) (mean, SD)^*^	11.6 (0.9)	12.2 (2)	0.16
BCTQ three months (function) (mean, SD)^#^	9 (1.1)	10.2 (2.1)	0.007^*^
HADS (D) pre (mean, SD)	2.5 (3.3)	1.7 (2.2)	0.75
HADS (A) one week (mean, SD)	1.4 (1.9)	1.1 (1.4)	0.70
HADS (D) one week (mean, SD)	0.8 (1.6)	0.7 (1.2)	0.56
Failures, anesthesia n (%)	0	5 (13.1)	0.02
Complications, clinical n (%)	2 (5)	6 (15.7)	0.14

^*^Statistically significant (P < 0.05); ^#^for the 3-month assessment, WALANT (n = 38) and IVRA (n = 34); IVRA = intravenous regional anesthesia; SD = standard deviation; pre = preoperative; BCTQ = Boston Carpal Tunnel Questionnaire; HADS (A) = Hospital Anxiety and Depression Scale - anxiety subscale; HADS (D) = Hospital Anxiety and Depression Scale - depression subscale.

### Drugs used in the first postoperative week

The WALANT group used 5.7 ± 9.81 dipyrone tablets in the first postoperative week, while the IVRA group used 10.8 ± 9.8 (P = 0.02). Regarding use of tramadol tablets, the WALANT group used on average 1.6 ± 3.3, while the IVRA group used 4 ± 8.0 (P = 0.066) (Table 3).

### Patient-reported function

The evaluations of symptoms and function through the Boston Carpal Tunnel Questionnaire (BCTQ) before and after surgery were similar between the groups (Table 3).

### In-hospital anxiety and depression

The Hospital Anxiety and Depression Scale (HADS) showed that there was no significant difference from before to after the surgery between the groups studied **(**Table 3**)**.

### Paresthesia after surgery

All the patients presented remission or significant improvement of paresthesia three months after the surgical procedure, without any difference between the groups (Table 3**)**.

### Complications and failures

Five cases of anesthetic failure were recorded in the IVRA group. Two patients presented intense pain at the time of the cutaneous incision and two patient presented intense pain at the site of the tourniquet, with all of them requiring anesthetic intervention for intravenous sedation. One patient did not present any venous access to the limb that was to be operated on, and local anesthesia was chosen. No failures were found in the WALANT group **(**Table 3**).**

The clinical complications included one case in the WALANT group of surgical wound dehiscence after a fall from the patient’s own height. This case evolved with healing after local care. “Pillar pain” occurred in three cases: one in the WALANT group and two in the IVRA group. All evolved with improved symptoms. One patient in the IVRA group with extreme pain returned for reevaluation in the emergency room, a few hours after hospital discharge, and required analgesia with morphine. Three patients had significant hematomas that needed postoperative clinical care **(**Table 3**).**

## DISCUSSION

The study groups were homogeneous and were compatible with the standard epidemiology of CTS.^21^ During the hospital stay, the pain measured on the visual analogue scale (VAS) was higher in the IVRA group in the immediate postoperative period and at 2 hours, 4 hours, 6 hours and 8 hours after surgery, with a statistical difference (P < 0.05). From the immediate postoperative period until the second hour after surgery, this difference was clinically relevant (> 2 points on the VAS), as indicated in the literature.^11^ This was possibly due to the rapid dissipation of the anesthetic and short latency of IVRA anesthesia, with early recovery of sensory and motor functions after release of the tourniquet, while the WALANT group presented low levels of pain at these times, probably due to the longer half-life of the anesthetic (around 3 to 5 hours).^22^^,^^23^

The difference in the mean times spent by the participants in the operating room was 12 minutes (higher in the IVRA group). This was due to the need for additional procedures, such as access to the limb to be operated and also the need to only release the tourniquet after a minimum safety time of 30 minutes after intravenous infusion of the anesthetic. The mean duration of surgery in the WALANT group was 2.1 minutes longer than in the IVRA group, possibly due to the need for detailed intraoperative hemostasis and due to the distortion of the anatomy resulting from the fluid and tissue edema in local anesthesia, a result that was in agreement with findings from other studies.^11^^,^^24^

The reported function (BCTQ), the anxiety and depression questionnaire (HADS) and the evaluation of the remission of paresthesia did not reveal any statistical difference between the groups, from before surgery to one month post-surgery. Thus, it could be inferred that the anesthetic techniques did not influence the final clinical result. We found that there was a statistical difference in the three-month assessment, but the numerical data suggested that it may not have been clinically relevant (9 versus 10.2 points).

The IVRA success rate reported in the literature is 96%-100%.^25^ We obtained a failure rate (13%) that was above that reported in the literature. However, in analyzing the previous studies individually we found several sources of bias. It was common for the authors to observe that some patients also received supplementary medication in varying doses, such as fentanyl and propofol. However, they considered that IVRA was successful due to avoidance of conversion to general anesthesia because of insufficient analgesia.^26^^,^^27^^,^^28^^,^^29^

All the results found in our study were in agreement with the results from a randomized trial among 24 patients with bilateral CTS who were operated on one hand using WALANT and on the contralateral hand using the IVRA method. The outcomes were pain, expectations and feelings about the reoperation. The conclusion from that trial was that local anesthesia offered a better intraoperative and postoperative experience in relation to pain, and that the patients had a broad preference for WALANT.^30^

Although our results clearly demonstrated the superiority of the WALANT method, our study had limitations because it was a single-group experience, which did not allow a definitive conclusion to be reached regarding this subject. Our study sample may not have had enough power for all the secondary outcomes and also for the baseline data. No cost-effectiveness approach was investigated, and it was not possible to estimate the amount of resources saved through the WALANT technique. Nonetheless, recent data from trigger finger release procedures demonstrated that WALANT was cost-effective, which makes us believe that this pattern may be the same for CTS.^31^

## CONCLUSIONS

The WALANT technique was more effective than IVRA in relation to pain control, operating room time, use of analgesic in the postoperative period and the failure rate, in open surgery for treating CTS.
